# Promoting healthy cardiovascular aging: emerging topics

**DOI:** 10.20517/jca.2022.27

**Published:** 2022-07-29

**Authors:** Zachary S. Clayton, Daniel H. Craighead, Sanna Darvish, McKinley Coppock, Katelyn R. Ludwig, Vienna E. Brunt, Douglas R. Seals, Matthew J. Rossman

**Affiliations:** Department of Integrative Physiology, University of Colorado Boulder, Boulder, CO 80309, USA.

**Keywords:** Hallmarks of aging, exercise, nutrition, oxidative stress, inflammation, endothelial function, arterial stiffness

## Abstract

The development of age-related cardiovascular (CV) dysfunction increases the risk of CV disease as well as other chronic age-associated disorders, including chronic kidney disease, and Alzheimer’s disease and related dementias. Major manifestations of age-associated CV dysfunction that increase disease risk are vascular dysfunction, primarily vascular endothelial dysfunction and arterial stiffening, and elevated systolic blood pressure. Declines in nitric oxide bioavailability secondary to increased oxidative stress and inflammation are established mechanisms of CV dysfunction with aging. Moreover, fundamental mechanisms of aging, termed the “hallmarks of aging” extend to the CV system and, as such, may be considered “hallmarks of CV aging”. These mechanisms represent viable therapeutic targets for treating CV dysfunction with aging. Healthy lifestyle behaviors, such as regular aerobic exercise and certain dietary patterns, are considered “first-line” strategies to prevent and/or treat age-associated CV dysfunction. Despite the well-established benefits of these strategies, many older adults do not meet the recommended guidelines for exercise or consume a healthy diet. Therefore, it is important to establish alternative and/or complementary evidence-based approaches to prevent or reverse age-related CV dysfunction. Targeting fundamental mechanisms of CV aging with interventions such as time-efficient exercise training, food-derived molecules, termed nutraceuticals, or select synthetic pharmacological agents represents a promising approach. In the present review, we will highlight emerging topics in the field of healthy CV aging with a specific focus on how exercise, nutrition/dietary patterns, nutraceuticals and select synthetic pharmacological compounds may promote healthy CV aging, in part, by targeting the hallmarks of CV aging.

## INTRODUCTION

Cardiovascular diseases (CVD) are the leading cause of morbidity and mortality in the US and most modern societies^[1,2]^. Advancing age is the primary non-modifiable risk factor for CVD and, as such, > 90% of CVD occur in midlife/older (ML/O) adults (i.e., adults aged ≥ 50 years). Importantly, a new epidemic of CVD is projected in the near future as a consequence of a demographic shift toward older populations in developed nations^[3,4]^. In the US alone, the number of older adults is expected to double by 2050^[4]^; without effective intervention, it is projected that 40% of adults in the US will have one or more forms of CVD by 2030^[3]^.

**Figure 1.**
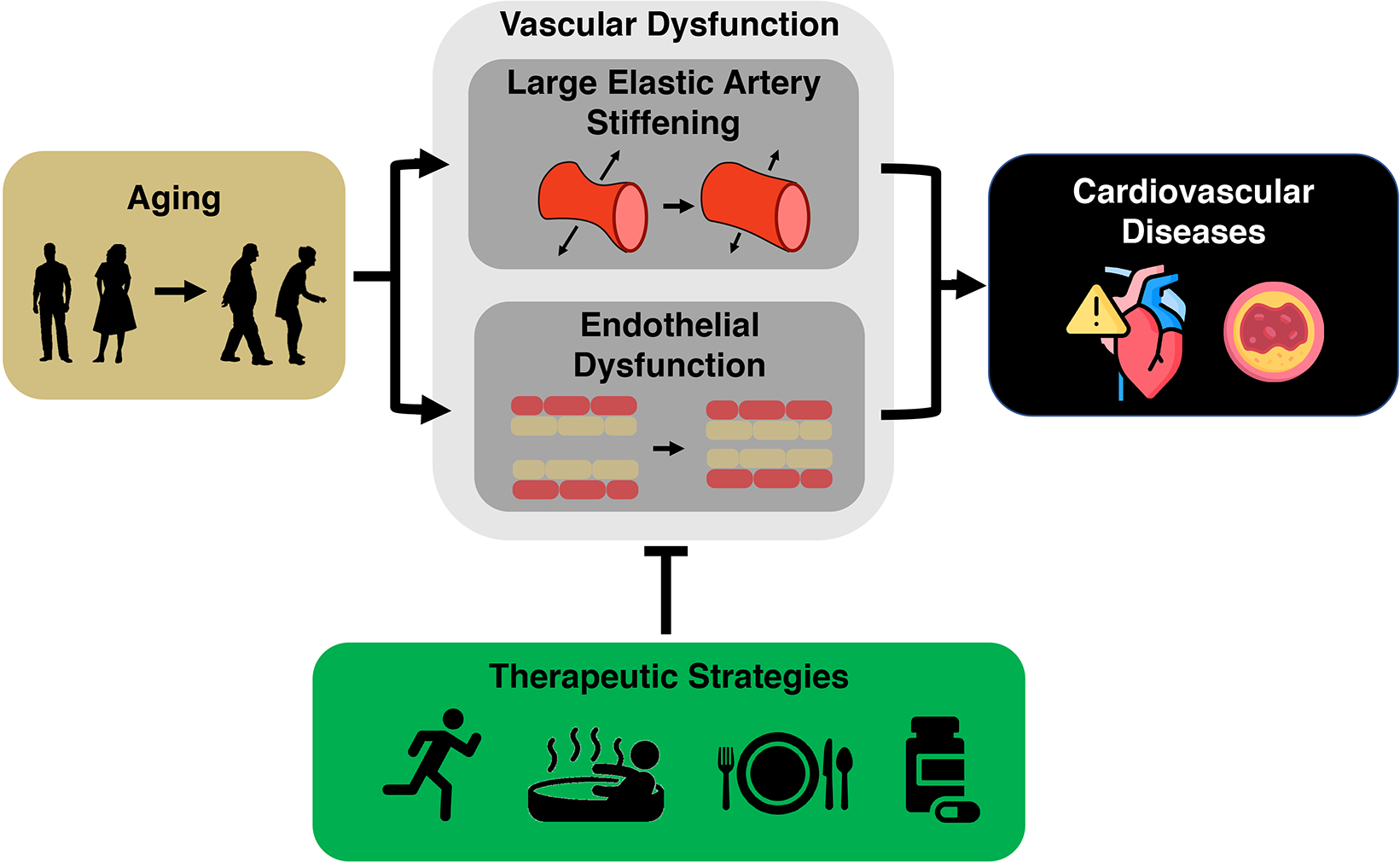
Cardiovascular aging and the need to develop effective therapeutic strategies. Aging is the primary risk factor for the development of cardiovascular diseases. This increase in risk is largely mediated by the development of vascular dysfunction, characterized primarily by large elastic artery stiffening and endothelial dysfunction. It is an important biomedical research priority to develop evidence-based therapeutic strategies to target these manifestations of vascular dysfunction to reduce the risk for cardiovascular diseases.

The key intermediate events linking aging with increased risk of CVD are the development of vascular dysfunction, including vascular endothelial dysfunction and stiffening of the large elastic arteries (i.e., the aorta and carotid arteries)^[5–7]^, and increased systolic blood pressure (SBP)^[8]^. Healthy lifestyle behaviors such as regular aerobic exercise and certain dietary patterns are considered “first-line” strategies to favorably modulate vascular function and SBP to reduce CVD risk with aging^[9]^. Although there are well-documented benefits of these behaviors, over 60% of older adults do not meet the recommended guidelines for exercise or consuming a healthy diet^[10,11]^. Thus, as we move toward an era of precision medicine and individualized treatments, it is a biomedical research priority to establish alternative and complementary therapeutic strategies for improving cardiovascular (CV) health with aging to provide options to those individuals for whom adherence to current guidelines may not be possible. To accomplish this goal, we need to establish an evidence base for alternatives to first-line strategies. Ideal alternatives are interventions that overcome barriers to performing regular exercise and eating a healthy diet (e.g., lack of time and education and/or limited access to gym facilities and healthy food options due mostly to a variety of socioeconomic inequities^[12–17]^). Time-efficient forms of aerobic exercise, “exercise-inspired” interventions (e.g., mild, controlled exposure to environmental stressors to promote physiological adaptations), or more adherable dietary practices hold promise in this regard. Moreover, an increased understanding of the mechanisms by which exercise and diet exert beneficial effects facilitates the identification of “therapeutic targets” for compounds (natural or synthetic) to recapitulate, in part, the effects of healthy lifestyle practices on vascular aging [Figure 1].

This review highlights emerging topics for the promotion of healthy CV aging, with a particular focus on vascular function, and select discussions on blood pressure (BP). Although important for CV aging, this review will not discuss age-related changes in cardiac function, as this topic has been reviewed elsewhere^[18]^. Furthermore, the majority of this review will discuss work conducted within our laboratory, in the area of CV aging, at the University of Colorado Boulder. Moreover, we have published previous reviews focused on therapeutic strategies for promoting healthy vascular aging^[9,19,20]^; thus, the present review will focus on *emerging* interventions being studied in our laboratory. Specifically, we summarize the features and mechanisms of vascular dysfunction with aging (reviewed in detail elsewhere^[21–23]^), discuss fundamental mechanisms of aging underlying CV aging, and provide an update on newly established therapeutic strategies that may promote CV health with advancing age, in part, by targeting these mechanisms. In addition, we highlight other developing concepts for achieving healthy CV aging, while also discussing research gaps and potential future directions.

## VASCULAR ENDOTHELIAL DYSFUNCTION

**Figure 2.**
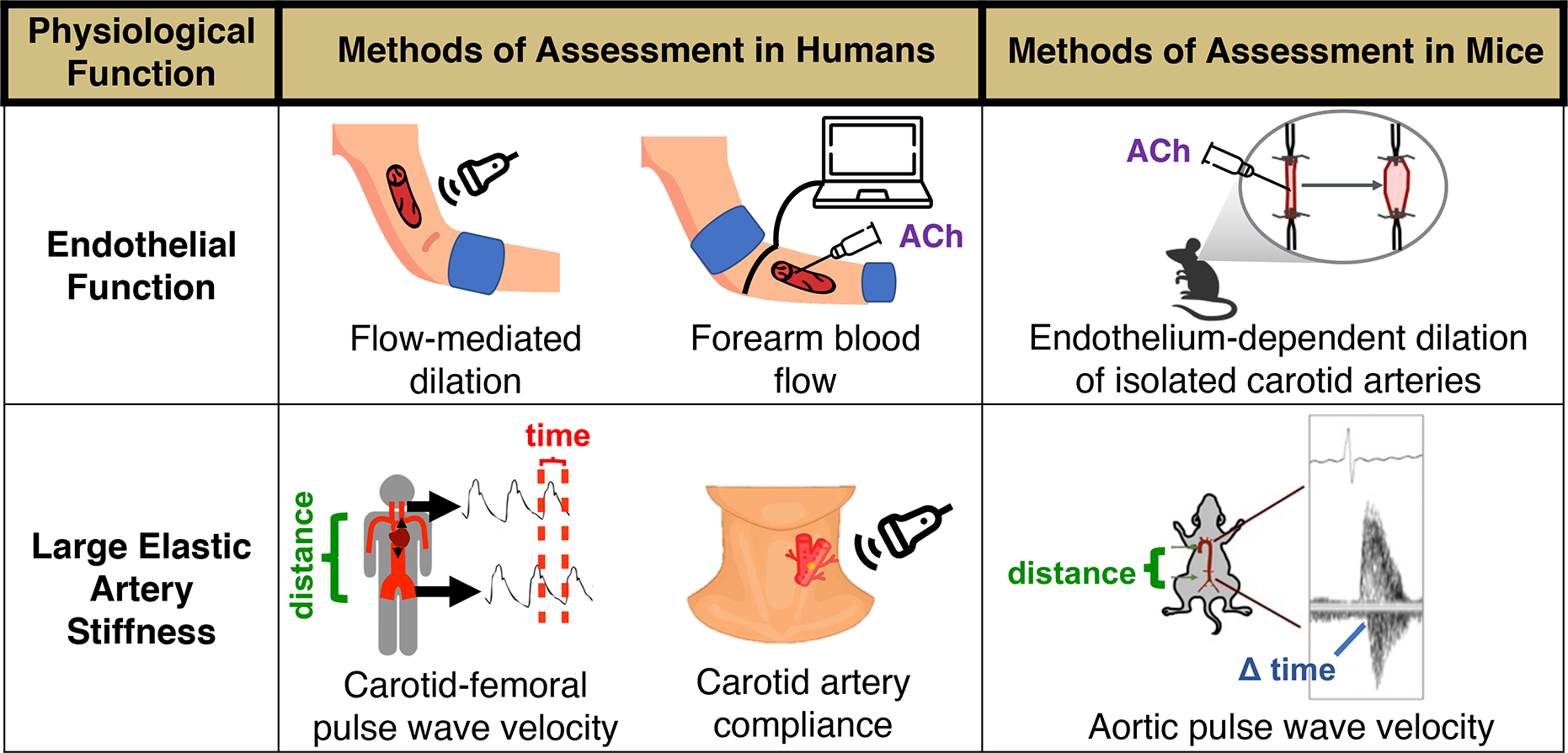
Methods for assessing endothelial function and arterial stiffness in humans and mice. Endothelial function can be determined through endothelium-dependent dilation. In humans, we assess this experimentally via brachial artery flow-mediated dilation (noninvasive gold standard of conduit artery function) or by assessing the forearm blood flow response to brachial artery infusion of acetylcholine (ACh) (resistance vessel function). In mice, we assess endothelium-dependent dilation by exposing isolated carotid arteries to increasing doses of a pharmacological stimulus such as ACh. We measure large elastic artery stiffness in humans via carotid-femoral pulse wave velocity (the gold-standard technique for assessing aortic stiffness), or by carotid artery compliance (a local measure of arterial distensibility). In mice, we measure arterial stiffness by aortic pulse wave velocity, measured between the aortic arch and abdominal aorta.

The vascular endothelium is a single layer of cells lining the lumen of blood vessels. The endothelium plays a critical role in the regulation of vascular tone and systemic blood blow, metabolism, thrombosis, immune system function and a variety of other processes^[24]^, in part through the production of vasodilatory and mostly vasoprotective molecule, nitric oxide (NO). Mechanical (i.e., blood flow-mediated shear stress) and chemical [e.g., acetylcholine (ACh) and insulin] stimuli elicit NO production in endothelial cells by activation of the enzyme, endothelial nitric oxide synthase (eNOS), which catalyzes the conversion of L-arginine and oxygen to NO. Endothelium-derived NO subsequently diffuses to vascular smooth muscle cells, where it activates an intracellular signaling cascade leading to vascular smooth muscle relaxation and vasodilation [endothelium-dependent dilation (EDD)]. In our laboratory, we assess the extent of NO-mediated EDD in humans by measuring the vasodilatory response of the brachial artery to hyperemia produced by temporary forearm ischemia (i.e., brachial artery flow-mediated dilation), which is considered the gold-standard noninvasive assessment of macrovascular (conduit artery) function^[24]^. In addition, we measure microvascular (resistance vessel) EDD via the forearm blood flow response to brachial artery infusion of ACh^[24]^. In rodents, flow-mediated dilation and the change in diameter of isolated arterial segments in response to pharmacological stimuli such as ACh can also be used to assess EDD^[25,26]^ [Figure 2]. Both conduit and resistance artery EDD are indices of endothelial health and independent predictors of CVD risk^[24,27–31]^ that decline with aging, in large part due to decreased NO bioavailability. As such, reduced conduit and resistance artery EDD are primary antecedents to overt CVD in older adults (i.e., directly increase the risk of developing atherosclerosis, coronary artery disease and occlusive stroke^[5]^).

## MECHANISMS OF ENDOTHELIAL DYSFUNCTION WITH AGING

Numerous molecular and cellular mechanisms underlie the adverse effects of aging on endothelial function. Of these, oxidative stress, characterized by an excess of reactive oxygen species (ROS) production (primarily superoxide) relative to endogenous antioxidant defenses, is a primary mechanism by which aging results in vascular dysfunction, largely via ROS-mediated reductions in NO bioavailabilty^[27,32–34]^. Excessive ROS in endothelial cells react directly with NO, leading to its deactivation and formation of peroxynitrite, another species of ROS. Furthermore, ROS can oxidize tetrahydrobiopterin, an essential co-factor for NO synthesis via eNOS, which results in eNOS uncoupling, whereby eNOS becomes a ROS generator, producing superoxide rather than NO^[35,36]^. Specific sources of vascular ROS with aging are increased NADPH oxidase activity^[32,37]^ and dysfunctional mitochondria^[38–41]^. Unchanged or reduced endogenous antioxidant enzyme defenses, including isoforms of superoxide dismutase (SOD)^[24]^, also contribute to this state of vascular oxidative stress.

Age-associated increases in chronic low-grade inflammation also play a prominent role in promoting endothelial dysfunction^[24,42]^. Pro-inflammatory mediators such as the transcription factor nuclear factor-κB (NF-κB) are increased with aging in endothelial cells (and other cell types) and contribute to impaired endothelial function, at least in part, by promoting increased oxidative stress^[43–45]^. Increased production of pro-inflammatory cytokines may also directly contribute to increased oxidative stress via inflammasome activation-mediated increases in mitochondrial ROS^[46]^.

## LARGE ELASTIC ARTERY STIFFNESS

The large elastic arteries (i.e., the aorta and carotid arteries) expand and recoil with each bolus of blood ejected from the left ventricle during systole. The processes of expanding and recoiling allow for dampening of the oscillatory pulse of blood that is ejected into the arterial system, aid in the propulsion of blood into the peripheral circulation, and help maintain perfusion of the heart during diastole^[25]^. The pulsatility-dampening effect of large elastic arteries is critical for reducing the transmission of harmful high pulsatile pressures to low-impedance, high flow sensitive organs such as the kidneys and the brain^[47]^. However, the large elastic arteries stiffen with advancing age, resulting in the ejection of blood into a stiffer aorta, which increases central and peripheral SBP as well as the work of the heart to overcome the resulting increase in afterload^[48]^. Moreover, the forward-moving pressure wave generated by the ejection of blood into the aorta travels at greater velocity along the stiffer arteries, which alters the timing of the pressure wave reflected by points of impedance in the arterial tree, such that the returning pressure wave reaches the heart during systole. The early return of the reflected pressure wave, in turn, further augments central SBP and afterload and negates the ability of the reflected wave to support perfusion of the heart during diastole, contributing to a decline in diastolic BP (DBP) with advancing age^[48–50]^. The change in timing of the reflected wave also results in a greater transmission of the forward-moving pressure wave to the microcirculation, which may damage small arterioles and capillaries leading to reduced blood flow and oxygen delivery to distal organs, such as the kidney and brain^[47]^.

Arterial stiffness can be assessed regionally by measuring the velocity of the arterial pressure pulse wave [pulse wave velocity (PWV)] traveling through a defined arterial segment. The most clinically significant expression of arterial stiffness is aortic stiffness^[51]^ and the gold-standard clinical measure of aortic stiffness is carotid-femoral PWV^[52]^; aortic stiffness can also be assessed by measuring aortic PWV in mice^[38,53,54]^ [Figure 2]. Similar to EDD, carotid-femoral PWV independently predicts CVD risk with aging^[55]^. A growing body of evidence also implicates aortic stiffening in the pathogenesis of Alzheimer’s disease^[56,57]^ and declines in cognitive function^[58–60]^, as well as decreases in renal function^[61–63]^ and glucose tolerance^[57,64,65]^, consistent with the notion of greater pulsatility transmission-related damage to these high-flow organs^[47]^. Arterial stiffness can also be assessed locally by measuring arterial distensibility, which is commonly assessed in the carotid artery as carotid artery compliance (inverse of stiffness) [Figure 2]. Carotid artery compliance decreases with advancing age^[66,67]^ and is independently (controlling for aortic stiffness) associated with an increased risk of CV events, particularly incident stroke^[57,68]^.

## MECHANISMS OF ARTERIAL STIFFENING

Stiffening of the large elastic arteries with advancing age is mediated by structural changes in the arterial wall as well as functional changes that increase vascular smooth muscle tone^[9,19]^. Structural changes include remodeling of the extracellular matrix (increased collagen deposition and elastin fragmentation and degradation) and formation of advanced glycation end products (AGEs), which increase stiffening by crosslinking structural proteins^[5,69,70]^. These structural changes that promote aortic stiffening are induced by mechanical events (i.e., repeated mechanical loading associated with cyclical changes in arterial pressure). In addition, oxidative stress may lead to arterial stiffening by increasing collagen deposition^[71]^. Excess oxidative stress and pro-inflammatory signaling also likely contribute to age-related arterial stiffening by increasing vascular smooth muscle tone, at least in part, by reducing the bioavailability of NO^[52,72]^. Greater renin angiotensin-aldosterone system signaling, sympathetic nervous system activity, and endothelin-1 system activation also contribute to increased vascular smooth muscle tone and arterial stiffness with aging^[73–75]^. Furthermore, augmented intrinsic stiffness of vascular smooth muscle cells may also play a role^[76]^.

## ABOVE-NORMAL SYSTOLIC BLOOD PRESSURE AND UNDERLYING MECHANISMS

Isolated systolic hypertension, elevated SBP but not DBP, is the most common type of hypertension in ML/O adults and is an independent predictor of CV-related morbidity and mortality^[77]^. Indeed, longitudinal data from the Framingham Heart Study, demonstrated that SBP continuously increases between 30 to 84 years of age (or older)^[50]^. However, DBP demonstrated a varying pattern with aging, increasing until age 50 and slowly reducing from ages 60 to 84 years, which ultimately leads to a steep, age-related rise in pulse pressure [SBP (−) DBP]^[50]^. These observations have since been corroborated in other large population longitudinal studies^[77–80]^.

The increase in SBP with aging is largely related to changes in arterial stiffness, as described above and in part, due to an increase in peripheral vascular resistance - the resistance in the circulatory system that is used to support BP; additionally, it is also induced by decreased baroreceptor sensitivity, increased activity of and responsiveness to the sympathetic nervous system, altered sodium metabolism, alterations in renin-angiotensin-aldosterone system signaling and decreases in NO bioavailability and endothelial function - reviewed in detail elsewhere^[81]^. Moreover, above-normal SBP also has been linked to increased oxidative stress and inflammation^[82]^.

## MECHANISTIC HALLMARKS OF CV AGING

**Figure 3.**
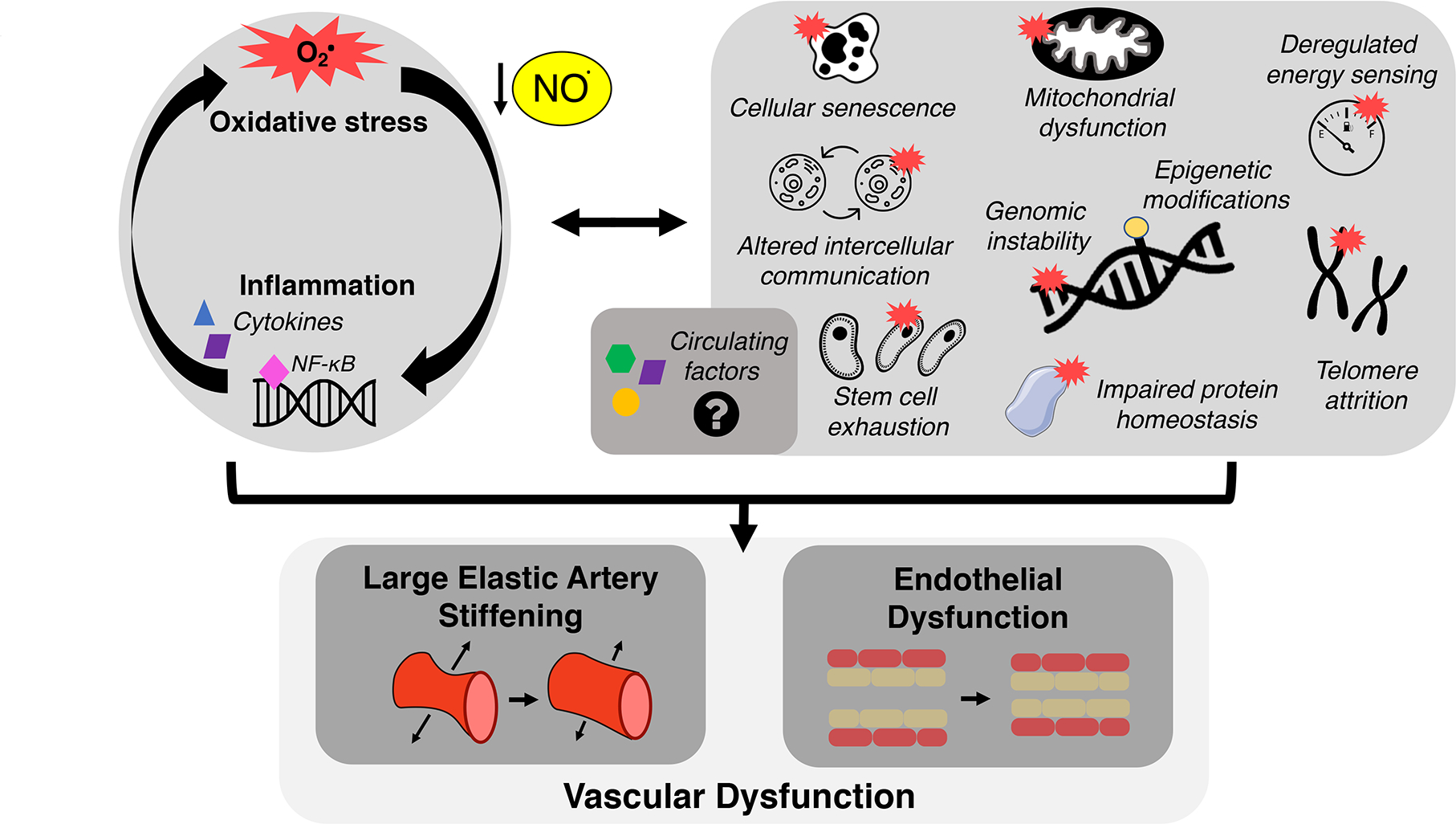
The hallmarks of cardiovascular aging. Increases in oxidative stress and chronic, low-grade inflammation leading to declines in nitric oxide (NO) bioavailability are established mechanisms of vascular dysfunction with aging. The hallmarks of cardiovascular aging, including cellular senescence and mitochondrial dysfunction, contribute to the development of vascular dysfunction in part by promoting these mechanisms. These hallmarks may also exert some of their effects by directly or indirectly influencing the circulating milieu (i.e., circulating factors).

In the field of biomedical research on aging, a collection of interconnected cellular-molecular events underlying the aging process have emerged, originally termed the “hallmarks” of aging^[83]^ [Figure 3]. Although the precise fundamental aging mechanisms comprising the hallmarks of aging are debated and evolving^[84,85]^, there is accumulating evidence supporting these hallmarks as primary contributors to CV dysfunction with aging^[21,22]^. Below, we will highlight particular hallmarks of CV aging studied in our laboratory - mitochondrial dysfunction, loss of protein homeostasis, deregulated nutrient sensing, and cellular senescence - as mechanisms that contribute to vascular dysfunction, at least in part by promoting oxidative stress, increasing chronic low-grade inflammation/pro-inflammatory signaling, and reducing NO bioavailability. Furthermore, we describe how these hallmarks may influence vascular function via their influence on the circulating milieu [Figure 3]. The focus of this section is on cross-sectional comparisons in young *vs.* older adults and preclinical proof-of-concept evidence establishing these hallmarks as mechanisms of CV aging, including studies using genetic manipulation and/or pharmacological approaches not suitable for translation to treating vascular dysfunction in older adults free from clinical disease. The translational evidence for targeting these hallmarks is presented in the “interventions to promote healthy CV aging” section of this review. Lastly, we outline how these hallmarks may serve as viable therapeutic targets for promoting healthy CV aging.

### Mitochondrial dysfunction

With advancing age, there is a marked reduction in vascular mitochondrial health/fitness^[38,86]^ characterized, in part, by increased mitochondrial DNA damage^[87]^, excess production of mitochondrial ROS^[40,54]^, and a decrease in mitochondrial antioxidant enzyme defenses^[40,88]^ (which we have previously reviewed in detail^[38]^). Others have shown that increased mitochondrial DNA damage directly contributes to age-associated increases in arterial stiffness^[87]^. We have shown that excess mitochondrial ROS is a mechanism underlying vascular dysfunction with aging^[40,89]^. Another known feature of mitochondrial health/fitness is mitochondrial quality control - the collection of molecular processes by which new, healthy mitochondria are produced (mitochondrial biogenesis) and damaged mitochondria are degraded by mitophagy (mitochondrial autophagy)^[38,90]^. We have shown that relative to young control mice, old mice have reduced markers of mitochondrial biogenesis^[90]^ and mitophagy^[90]^ in arteries, indicative of impaired vascular mitochondrial quality control. The age-related changes in mitochondrial quality control markers were associated with greater vascular mitochondrial ROS bioactivity and marked a lower abundance of manganese (Mn)SOD, the primary isoform of SOD in the mitochondria^[90]^. Taken together, these data suggest that impaired mitochondrial health/fitness may be a mechanism of vascular aging, at least in part by promoting excess ROS.

An additional manifestation of mitochondrial dysfunction that contributes to age-related vascular dysfunction is a decline in mitochondrial stress resistance, i.e., the ability to maintain function when challenged with external stressors^[38]^. We first sought to determine the role of mitochondrial fitness in mediating age-related vascular dysfunction by administering the mitochondrial stressor rotenone or a simulated Western diet (e.g., high palmitate + high glucose) to isolated carotid arteries from young and old mice, and subsequently assessing endothelial function^[86]^. We found that these mitochondrial stressors further reduced endothelial function in old mice (i.e., to a greater extent than the reduction in EDD in old vs young mice without exposure to the stress), while there was no effect on young mouse vessels. Furthermore, the simulated Western diet-associated reduction in EDD was prevented in the presence of a mitochondrial-targeted antioxidant, demonstrating that Western diet-induced increases in mitochondrial ROS are responsible for the stressor-associated reduction in EDD in old mice^[86]^. These observations suggest that age-associated declines in mitochondrial stress resistance contribute to vascular dysfunction with aging.

### Loss of protein homeostasis

Similar to the process of mitochondrial quality control (described above), protein homeostasis is a process critical for the stabilization of correctly folded proteins, and proper degradation of proteins by the lysosome^[83]^. A principal proteolytic system implicated in protein quality control is the autophagy-lysosomal system^[83]^, which is markedly reduced in the aging vasculature^[21,22]^. This system operates either by delivering damaged proteins, macromolecules and organelles to a lysosomal receptor (chaperone-mediated autophagy) or via the formation of autophagosomes, specialized double-membrane vesicles that envelop target organelles/macromolecules and later fuse with a lysosome (macroautophagy)^[91]^. In a series of reverse translational studies (humans to mice to cell cultures), we established reduced vascular autophagy as a mechanism underlying vascular aging. We first demonstrated that age-related endothelial dysfunction was associated with reduced markers of autophagy in endothelial cells sampled from arteries in ML/O *vs.* young adults^[92]^. These same proteins were also reduced in the vasculature of old mice, which was associated with endothelial dysfunction, reduced NO bioavailability, elevated vascular ROS bioactivity and inflammation, and excess tonic ROS-related suppression of endothelial function^[92]^. Lastly, we found that inhibition of autophagy reduced NO production in cultured endothelial cells^[92]^. Collectively, these findings highlight reduced autophagy as a mechanism of age-related vascular dysfunction.

### Cellular senescence

Cellular senescence is a state of mostly permanent cell cycle arrest, which at a basal state (i.e., at a young age), plays a critical role in many physiological processes including wound healing^[93]^ and inhibiting potential cancer progression^[94]^. However, with advancing age, senescent cells accumulate in a variety of tissues and this accumulation has been implicated in numerous age-related diseases^[95]^, including CVD (as reviewed elsewhere^[96]^). Senescent cells secrete a number of pro-inflammatory molecules and growth factors. This “secretome” is collectively referred to as the senescence-associated secretory phenotype (SASP) and is thought to largely mediate the pathological effect of senescent cells.

In support of a role for cellular senescence in age-associated vascular dysfunction, we demonstrated that ML/O adults had higher levels of endothelial cell senescence relative to young adults, and that the level of endothelial cell senescence was inversely associated with endothelial function^[97]^. Subsequently, we aimed to determine the cause-and-effect role of cellular senescence in mediating age-associated vascular dysfunction by clearing senescent cells in old mice. Clearance of senescent cells selectively improved vascular function in old mice, without effect in young mice, suggesting that cellular senescence is a fundamental mechanism underlying endothelial dysfunction and arterial stiffness with aging^[98,99]^. Furthermore, our results suggest that clearance of senescent cells in old animals improves NO-mediated EDD and ameliorates oxidative stress-related suppression of endothelial function, demonstrating that senescent cells mechanistically contribute to excess vascular ROS with aging^[98,99]^. In addition, our observations suggest that the SASP may be a mechanism by which cellular senescence causes age-related vascular dysfunction^[99]^. Overall, these results show that cellular senescence and the SASP may be viable therapeutic targets for treating vascular dysfunction with aging.

### Deregulated nutrient sensing

Several key energy- and nutrient-sensing pathways responsible for maintenance of overall cellular homeostasis become deregulated with aging and have been implicated in the development of age-related declines in physiological function. Well-established energy- and nutrient-sensing pathways that become deregulated include: (1) reduced abundance and activation of sirtuin 1 (SIRT-1); (2) increased abundance and activation of the mammalian target of rapamycin (mTOR); and (3) reduced activation of adenosine monophosphate-activated protein kinase (AMPK).

SIRT-1: SIRT-1, a nicotinamide adenine dinucleotide (NAD^+^)-dependent histone deacetylase, responds to low energy states in the cell and activates energy preserving pathways^[100]^. SIRT-1 activity has been shown to decline with aging, which has been linked to physiological dysfunction^[101]^. In terms of vascular aging, in a series of translational mechanistic studies (mice to people), we found that *ex vivo* administration of a SIRT-1 activator (Sirtinol) to carotid arteries from old mice normalized endothelial function back to levels observed in young animals^[101]^. We also observed that, in ML/O *vs.* young adults, lower endothelial function was associated with a reduction in the abundance of SIRT-1 in endothelial cells sampled from arteries^[101]^. Next, we sought to determine a causal role for SIRT-1 as a mediator of vascular dysfunction *in vivo* via oral administration of the SIRT-1 activator SRT1720 to young and old mice. We found that SRT1720 could reverse age-related endothelial dysfunction (back to young levels), in part, by increasing NO bioavailability and ameliorating excess oxidative stress and inflammation. These findings were associated with increased SIRT-1 activation (assessed via acetylation status of proteins downstream of SIRT-1) in the vasculature^[102]^.

Age-associated declines in the bioavailability of the substrate for SIRT-1, NAD^+^, are also thought to contribute to age-related CV dysfunction^[21]^. Although the adverse effects of declines in NAD^+^ levels have most commonly been attributed to associated reductions in SIRT-1 activity, NAD^+^ is involved in numerous biological processes and there is some evidence that declines in NAD^+^ levels may elicit SIRT-1-independent effects on physiological dysfunction with aging^[103]^. Regardless, the decline in NAD^+^ bioavailability is hypothesized to occur in two primary ways: (1) a reduction in the NAD^+^ salvage pathway (the primary means of NAD^+^ biosynthesis^[104]^); and (2) an increase in the NAD^+^ consumption pathway (the primary means of NAD^+^ degradation^[105,106]^). Our translational research (mice to humans) focused on restoring NAD^+^ levels with aging using dietary supplementation with NAD^+^ precursors is discussed in the nutraceuticals section below.

mTOR: mTOR is a nutrient-sensing protein that is increased during times of energy surplus (i.e., increased levels of NADH relative to NAD^+^) to promote growth and cellular replication^[107]^. mTOR is increased with aging in a variety of cells/tissues and reducing mTOR, either genetically or pharmacologically, extends lifespan and increases markers of healthspan (i.e., the period of life free of major chronic diseases and disability) in a variety of species. Elevated mTOR is also associated with CV dysfunction, as reviewed elsewhere^[107]^. We have previously shown that mTOR is increased in the vasculature of old mice, which was related to endothelial dysfunction and arterial stiffness^[108]^. Next, we aimed to specifically determine the role of mTOR in regulating vascular dysfunction with aging, via the administration of rapamycin - a well-established inhibitor of mTOR. We found that chronic rapamycin treatment could reverse (back to young levels) age-related arterial stiffening and endothelial dysfunction, the latter as a result of increasing NO bioavailability and ameliorating excess vascular oxidative stress^[108]^. These changes were associated with reduced vascular mTOR activation [assessed via phosphorylation of S6 kinase and activation of AMPK (a negative regulator of mTOR^[109]^)]^[108]^.

AMPK: AMPK is a kinase that increases during low energy states - e.g., increased ratio of AMP to ATP, and is activated via ATP and ADP catabolism. As previously mentioned, AMPK activation is a negative regulator of mTOR. Thus, it is plausible that direct activation of AMPK may improve vascular function with aging, possibly via suppression of mTOR. Indeed, we found that vascular AMPK activation is lower in old *vs.* young animals^[110]^. Thus, to directly determine the role of AMPK in vascular dysfunction with aging, we administered aminoimidazole carboxamide ribonucleotide (AICAR; a well-established AMPK activator) to young and old animals and found that AICAR could ameliorate endothelial dysfunction by restoring NO bioavailability in old mice as a result of reducing tonic excess vascular oxidative stress, and that these changes were associated with greater vascular AMPK activation (assessed via phosphorylation of AMPK)^[110].^

Together, this work establishes the role of alterations in energy sensing pathways in vascular aging.

### Circulating factors

The idea that various bloodborne factors in circulation are altered with advancing age and disease, and that these “circulating factors” contribute to declines in physiological function, has been a longstanding concept in the field of aging biology, and an emerging topic in CV aging. Changes in circulating factors with aging are likely caused by and are a consequence of impairments in the cellular and molecular process comprising the hallmarks of aging, including those discussed above (e.g., cellular senescence and the SASP and alterations in intercellular communication^[83]^). The concept of circulating factors was brought about by seminal heterochronic parabiosis experiments conducted in the 19th century^[111]^. In these experiments, two mice were surgically joined, such that they develop a shared circulation that allows for continuous exchange of soluble factors at physiological levels through their common circulatory system^[112]^. This experimental approach has been used to demonstrate that aging phenotypes can be transferred via circulation - e.g., cardiac dysfunction^[113]^, cognitive decline^[114]^, and endothelial dysfunction^[115]^ - demonstrating that circulating factors may be crucial mechanisms or mechanistic intermediates by which age-related physiological dysfunction manifests.

Comparing young versus ML/O adults, we have shown that circulating (plasma) proteomic signatures change with advanced age and are associated with central indices of CV aging (e.g., SBP and endothelial function)^[116]^. Using a similar study design, we have demonstrated that metabolomic signatures can predict biological aging^[117]^, indices of CV aging^[118]^, and potentially the response to an intervention that increased endothelial function and reduced arterial stiffness in ML/O adults^[119]^. Subsequent studies demonstrated that treatment with circulating factors (via plasma exposure to endothelial cells in culture) collected from old mice that have undergone a healthy aging intervention can mediate positive changes in endothelial cell heath - i.e., greater endothelial cell NO production and lower ROS (versus cells treated with plasma from mice treated with placebo)^[120]^. We have also demonstrated that treatment of endothelial cells in culture with plasma collected from ML/O adults after *vs.* before CV health-promoting interventions can increase endothelial cell NO production and reduce ROS bioactivity, demonstrating a role of alterations in circulating factors in modulating endothelial function^[121,122]^.

Determining the specific circulating factors responsible for driving aging phenotypes is a compelling question in biomedical aging research. Putative circulating factors associated with CV health include, but are not limited to: (i) DNA (e.g., mitochondrial DNA^[123]^); (ii) RNAs (e.g., micro RNAs^[124]^, long non-coding RNAs^[125]^, circular non-coding RNAs^[125]^); (iii) growth factors (e.g., growth differentiation factor 11^[113]^ and 15^[126,127]^); (iv) plasma proteins (e.g., C-reactive protein^[128]^ and pro-inflammatory cytokines^[129]^), lipids^[130]^ and metabolites^[131,132]^; (v) endothelial cell-derived proteins (e.g., von Willebrand factor, chondroitin sulfate synthase 3, transient receptor potential cation channel subfamily C member 6^[133]^); and (vi) the SASP^[134]^. Of note, the potential role of the latter mechanism illustrates the potential overlap among “distinct” aging hallmarks (i.e., cellular senescence and altered intercellular communication^[83]^). Elucidating novel circulating mediators of vascular dysfunction with aging is an important goal of future research, which may be addressed with high-throughput “-omics”-based approaches (e.g., transcriptomics, proteomics, metabolomics, or any combination of these approaches, termed “multi-omics”). Determining the direct/mechanistic role of *specific* circulating factors in modulating CV health with aging will be discussed further in the research gaps/future directions section.

## INTERVENTIONS TO PROMOTE HEALTHY CV AGING

In the subsequent sections of this review, we highlight the various therapeutic strategies/interventions we have studied in our laboratory to promote healthy CV aging and how these interventions may elicit beneficial effects, including targeting the aforementioned hallmarks of CV aging. We will specifically focus on the roles of (i) exercise; (ii) dietary patterns; and (iii) nutraceuticals and select synthetic pharmacological agents.

### Exercise training

**Figure 4.**
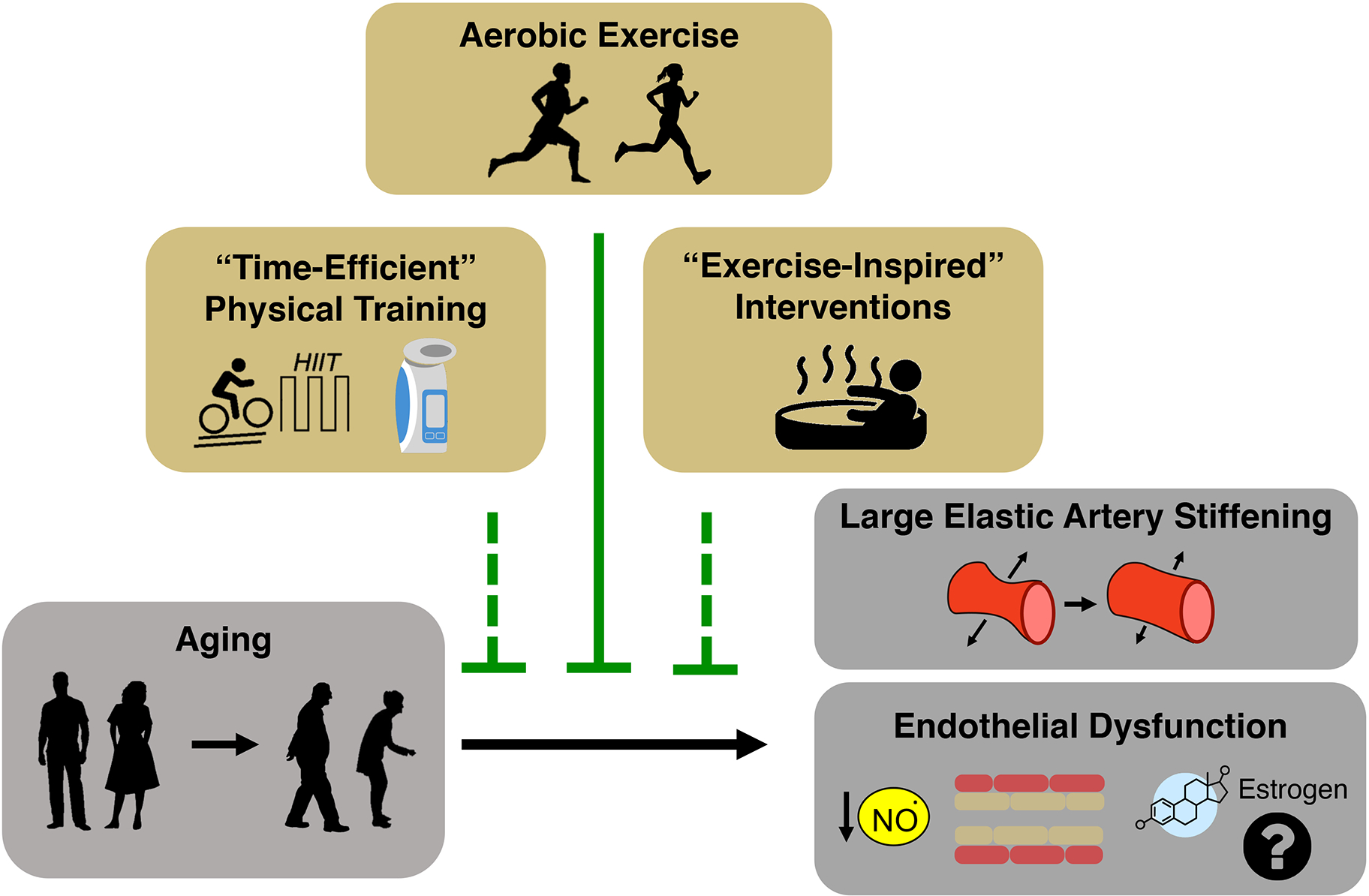
Aerobic exercise for improving CV health with aging - alternative strategies. Regular aerobic exercise is the most well-established lifestyle intervention (solid line) for improving cardiovascular function with aging. Despite this, many midlife and older adults do not meet physical activity guidelines, leading to a biomedical research need to develop alternative interventions (dashed lines) such as “time-efficient” physical training [i.e., high-intensity interval training (HIIT) and inspiratory muscle strength training] and “exercise-inspired” interventions (i.e., passive heat therapy). In addition, more research needs to be done to determine the role of sex hormones (i.e., estrogen) in mediating the aerobic exercise-induced improvements in endothelial function.

Exercise involves any structured form of physical activity performed to improve health and/or physical fitness. This section first discusses guideline-recommended forms of physical activity for older adults, such as conventional aerobic exercise and resistance training, for improving CV aging. We then discuss emerging time-efficient exercise modalities and “exercise-inspired” interventions as promising new strategies for improving CV health that may work through novel physiological mechanisms and overcome barriers preventing adherence to physical activity guidelines [Figure 4].

#### Aerobic exercise

##### Endothelial function

Aerobic exercise training is the most well-known and well-studied healthy lifestyle intervention for improving CV function with aging^[48,135,136]^. Aerobic exercise has consistently been shown to improve endothelial function in ML/O men. In cross-sectional comparisons, endurance-trained ML/O men have better endothelial function relative to their untrained peers^[33,137]^, as assessed by flow-mediated dilation^[33]^ and an increase in forearm blood flow in response to brachial artery infusion of ACh^[137]^. We have shown that greater endothelial function in endurance-trained ML/O is due to greater NO bioavailability^[48]^ and lower oxidative stress^[33,88]^ and inflammation^[44,138]^. In line with findings from cross-sectional studies, aerobic exercise training improves endothelial function in previously sedentary older men^[137,139]^, as a result of ameliorating excess oxidative stress-mediated suppression of endothelial function^[33]^.

To further determine the mechanisms by which aerobic exercise training improves endothelial function, we performed a series of reverse translational studies in male mice given access to voluntary exercise (running wheels). This study revealed that voluntary wheel running, when introduced in late life, could treat endothelial dysfunction with aging by increasing NO bioavailability and ameliorating whole cell^[37,140]^, NADPH oxidase-mediated^[37]^, and mitochondrial-specific^[86]^ oxidative stress. Furthermore, we found that voluntary wheel running prevented the exacerbation of endothelial dysfunction induced by short-term consumption of a Western diet (high fat and high sugar) during old age^[140]^. Next, we conducted a lifelong study in mice to determine if voluntary wheel running (a mouse model of voluntary aerobic exercise) throughout the lifespan could prevent age-related reductions in endothelial function, in the settings of both chronological aging and accelerated aging induced by consumption of a Western diet. We found that voluntary wheel running preserved endothelial function throughout the lifespan in both models of aging as a result of preserving NO bioavailability and preventing excess whole cell and mitochondria-specific ROS production^[54]^.

Interestingly, aerobic exercise training has not consistently been shown to improve endothelial function in previously sedentary estrogen-deficient postmenopausal women^[141]^ - i.e., postmenopausal women not taking hormone replacement therapy, which represents ~90% of postmenopausal women in the US^[142]^. Specifically, our^[139,143]^ and other^[144,145]^ laboratory’s aerobic exercise intervention studies fail to show an improvement in endothelial function following aerobic exercise training in these women. To demonstrate that these findings were unrelated to exercise dose, we showed that even in estrogen-deficient postmenopausal Masters endurance athletes who achieved very high volumes of aerobic exercise and had higher aerobic fitness, endothelial function was similar to their sedentary peers^[146]^. The lack of improvement in measures of endothelial function is thought to be due, at least in part, to the antioxidant effects of estrogen, as aerobic exercise training with concurrent estrogen replacement therapy (i.e., oral and transdermal estradiol) can improve endothelial function in these women via suppression of oxidative stress^[141,143]^. Future work, including studies in humans and preclinical animal models of menopauser^[147,148]^, is warranted to further investigate the mechanisms by which voluntary aerobic exercise may influence vascular endothelial function and how this response differs by sex and/or gende.

##### Arterial stiffness

In contrast to the sex-specific effects of aerobic exercise on vascular endothelial function, both ML/O adult men and women who are aerobically trained have lower arterial stiffness compared with age-matched sedentary peers^[66,67,149–153]^, which appears to be, in part, related to lower vascular inflammation^[138]^. Furthermore, we^[67]^ and others^[149,154,155]^ have shown that aerobic exercise training can decrease carotid artery stiffness in previously sedentary ML/O men and women. Longer aerobic exercise interventions and/or higher intensity exercise might be needed to reduce aortic stiffness, as cross-sectional studies suggest a dose-dependent effect^[156]^.

Next, we performed reverse translation experiments in mice to determine potential mechanisms by which aerobic exercise reduces arterial stiffness with aging. We found that voluntary wheel running, when introduced in late life, can reduce/treat arterial stiffness in old mice as measured by aortic PWV, which occurred as a result of reduced aortic intrinsic mechanical wall stiffness (an assessment of the material stiffness of the vessel wall) and was associated with lower aortic oxidative stress^[157]^. Next, in the same lifelong mouse study described above, we sought to determine if voluntary wheel running throughout the lifespan could prevent aortic stiffening with chronological aging and accelerated aging induced by Western diet feeding. Indeed, lifelong voluntary wheel running could fully prevent the progression of aortic stiffening in both models of aging due to preventing increases in aortic intrinsic mechanical wall stiffness and vascular inflammation^[54]^.

##### Blood pressure

In addition to improvements in endothelial function and arterial stiffness, a large body of literature has demonstrated that guideline-based aerobic exercise in ML/O men and women lowers resting SBP by 2–8 mmHg on average, with the largest reductions being shown in those with the highest baseline SBP (i.e., stage 2 hypertension, baseline SBP ≥ 140 mmHg^[158,159]^).

##### Mechanisms of action

Much of the previous work investigating the cellular-molecular mechanisms by which aerobic exercise improves CV function with aging has largely focused on oxidative stress and inflammation^[48]^. However, aerobic exercise is also thought to impart some benefits to vascular function by modulating fundamental aging mechanisms (the hallmarks of aging discussed above). For example, we have shown in mice that aerobic exercise initiated in late life can improve vascular mitochondrial health^[86]^ and that lifelong aerobic exercise can prevent age-related reductions in mitochondrial health in the vasculature^[54]^. Furthermore, aerobically exercise-trained older adults have lower markers of endothelial cell senescence relative to their sedentary age-matched peers, and the differences in cellular senescence are associated with endothelial function (e.g., higher endothelial cell senescence is associated with lower brachial artery flow-mediated dilation)^[97]^. As such, the ultimate effect on vascular function of modulating the hallmarks of aging with aerobic exercise may be mediated via suppression of oxidative stress and inflammation or bi-directional effects.

##### Resistance training

Along with aerobic exercise, regular resistance training is recommended in physical activity guidelines for older adults^[160,161]^, given the clinical importance of maintaining strength, power, and muscle mass to prevent sarcopenia and preserve physical function. However, unlike aerobic exercise, resistance training may not consistently improve endothelial function or reduce arterial stiffness and BP. In fact, ML/O adults who perform vigorous resistance training alone tend to exhibit similar or greater arterial stiffness (e.g., lower carotid artery compliance) and higher BP (e.g., carotid artery systolic pressure) compared to inactive peers^[162]^. However, there is evidence to suggest that a resistance training intervention can lower SBP in ML/O hypertensive women^[163]^. Training regimens that include both aerobic and resistance exercise training reduce age-related large elastic artery stiffening^[164]^. Interestingly, the order one performs resistance exercise relative to aerobic exercise may be important, as aerobic exercise only reduces arterial stiffness when performed after resistance exercise, but not before^[165]^. In terms of the influence of resistance training on endothelial function, flow-mediated dilation has shown to increase (improve) following resistance training in young healthy adults and patients with CV and metabolic diseases^[166]^, but there is minimal information in the published literature describing the influence of resistance training on endothelial function in ML/O adults. As such, future randomized-controlled trials (RCTs) are necessary to determine the effect of resistance training on vascular function with aging before it can be recommended as a lifestyle strategy for promoting healthy CV aging.

##### Time-efficient forms of physical training

Despite the well-recognized benefits of physical activity, particularly aerobic exercise, for CV health, many ML/O adults do not achieve the physical activity levels needed to meet guidelines for physical activity (i.e., 150 minutes/week moderate-intensity or 75 minutes/week vigorous-intensity aerobic exercise). A significant barrier to ML/O adults meeting physical activity guidelines is a perceived lack of time^[13]^. This barrier is a particularly important issue for many midlife men and women, because this is the period of life in which both family responsibilities and professional opportunities tend to peak^[161,167]^. Thus, there is an obvious need to develop time-efficient evidence-based alternative approaches to conventional (continuous) aerobic exercise training for maintaining CV health with aging. Interventions that fill this gap would have great potential for promoting adherence while simultaneously improving CV function.

Considering these criteria, high-resistance inspiratory muscle strength training (IMST)^[122]^ and interval-based aerobic exercise (i.e., high-intensity interval training)^[135]^ represent two encouraging forms of time-efficient physical training that may improve CV function in ML/O adults. In addition, the concept of “exercise snacks” (brief isolated bouts of vigorous exercise performed over the course of the day with the goal of reducing sedentary time) and vigorous intermittent lifestyle physical activity (unstructured vigorous-intensity physical activities that occur as part of daily living) - may represent viable and time-efficient methods to improve CV function with aging^[168,169]^. Current evidence on both high-resistance IMST^[170,171]^ and interval-based aerobic exercise^[135]^ for improving CV aging has been reviewed recently. We recently completed a randomized, double-blind, sham-controlled, parallel-design pilot clinical trial on high-resistance IMST. We found that ~5 min per day of high-resistance IMST (30 breaths per day at 55%–75% maximal inspiratory pressure, 6 days per week, for 6 weeks) was safe, promoted excellent adherence (~95% of all prescribed training sessions completed), decreased SBP by 9 mmHg and increased vascular endothelial function by ~45% in ML/O adults with above-normal initial SBP^[122]^. In contrast to conventional aerobic exercise, vascular endothelial function was improved in the estrogen-deficient postmenopausal women who performed IMST. The CV benefits in this pilot trial were associated with reductions in oxidative stress and chronic low-grade inflammation, and alterations in circulating factors that improved endothelial cell function.

We and our collaborators are now extending these findings on high-resistance IMST with appropriately-powered clinical trials in ML/O adults with above-normal SBP (NCT04848675), estrogen-deficient postmenopausal women (NCT05000515), patients with obstructive sleep apnea (NCT04932447^[172]^), and patients with chronic kidney disease (CKD) (NCT04911491).

##### Passive heat therapy: an exercise-inspired intervention

Interventions that mimic the acute CV response to traditional aerobic exercise (e.g., increased heart rate, peripheral vasodilation, anterograde vascular shear stress, cardiac output, etc.), without the associated physical demands (i.e., joint loading and coordination), may also represent a novel approach to improving CV health in ML/O older adults. An emerging “exercise-inspired” intervention for improving CV health is passive heat therapy, which has been extensively reviewed elsewhere^[173]^. In brief, the documented effects of passive heat therapy on the CV system include increases in conduit and resistance artery endothelial function, NO bioavailability, angiogenesis and abundance of heat shock proteins (known to be both anti-oxidative and anti-inflammatory, and assist in maintaining protein homeostasis), and decreases in vascular resistance, autonomic tone, and SBP^[174–180]^. We have preliminary data from a pilot clinical trial to suggest that chronic passive heat therapy (thirty 60-min sessions of 40.5 °C water immersion), relative to a sham condition (thirty 60-min sessions of 36 °C), can increase NO-mediated endothelial function and reduce PWV and SBP (~10 mmHg) in ML/O adults^[177]^. There is evidence to suggest that circulating factors (e.g., serum) from young sedentary adults that have undergone chronic passive heat therapy can improve vascular cell health^[176,178]^; however, this remains to be determined in ML/O adults. We are now translating our findings from our initial pilot study and conducting a large-scale multi-year randomized-controlled trial (RCT) with heat therapy in ML/O adults (NCT05300971).

### Nutrition - healthy dietary patterns

#### Dietary restriction

Caloric restriction (CR) - reducing total caloric intake (by ~30%–40%) while maintaining adequate micronutrient consumption - is one of the most well-studied and documented dietary patterns that can favorably influence the CV system^[181]^. We have shown that short-term (8 weeks) CR in mice, initiated in late life, can reverse/treat vascular endothelial dysfunction by increasing NO bioavailability and reducing oxidative stress, which was associated with an upregulation of SIRT-1 activity in the vasculature^[182]^. Furthermore, we have shown that lifelong CR in mice can *prevent* endothelial dysfunction, arterial stiffening and increases in SBP, in part, by preventing excess oxidative stress, and preserving NO bioavailability and arterial wall structural proteins (e.g., collagen and elastin). Furthermore, we found that lifelong CR could preserve SIRT-1 and mTOR abundance and/or activity (no difference *vs.* young controls), suggesting that CR may preserve CV function throughout the lifespan, in part, by targeting the aging hallmark “deregulated nutrient sensing”^[183]^. In addition, CR may also improve CV health with aging by increasing autophagy, suggesting CR may also target the aging hallmark “impaired proteostasis”^[184]^.

**Figure 5.**
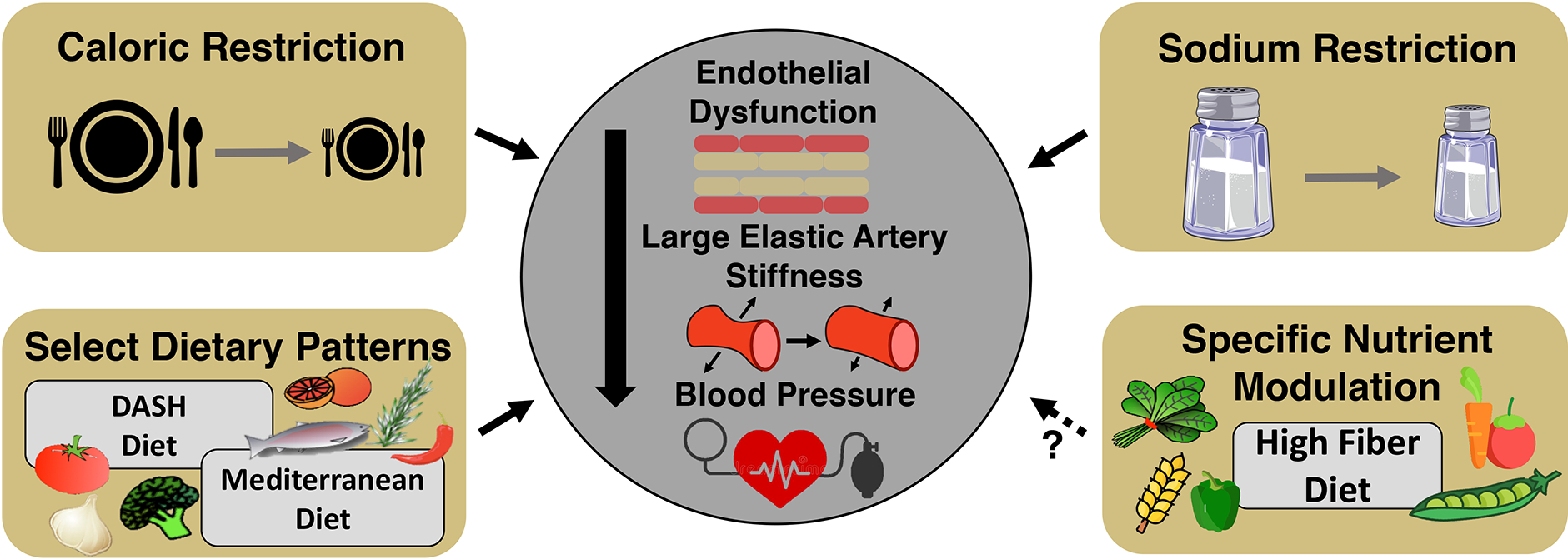
Nutrition and dietary patterns to improve cardiovascular function with aging. Select nutrients and dietary patterns have been shown to target manifestations of age-related cardiovascular dysfunction. It is well established that caloric (dietary) restriction and sodium restriction increase endothelial function and lower large elastic artery stiffness and blood pressure. Specific dietary patterns, such as the Dietary Approaches to Stop Hypertension (DASH) and Mediterranean diets, have been shown to improve blood pressure, but more studies need to be conducted to assess the effects of these dietary patterns in improving overall cardiovascular function in midlife/older adults. Recent evidence points to high soluble fiber as a possible nutrient in conferring vascular benefits. Subsequently, specific nutrient modulation of high fiber diets may be an effective and safe dietary pattern to improve overall vascular function in midlife/older adults.

Similar to CR, we have shown that diet-induced weight loss (consuming a diet designed to reduce bodyweight by 10%) in young and older overweight/obese adults can improve conduit and resistance artery endothelial function and that improved endothelial function was associated with reduced oxidative stress [assessed via circulating oxidized Low-density lipoprotein Cholesterol (LDL-C)]^[185]^. Furthermore, it has been shown that diet-induced weight loss in ML/O adults can reduce arterial stiffness and SBP (7–8 mmHg)^[186,187]^ [Figure 5]. Collectively, these results suggest that CR-mediated weight loss may be an effective dietary approach for improving CV health with aging.

Although most of the beneficial effects of CR are thought to be mediated by weight loss, CR also activates energy sensing pathways during the fasting period, leading to the idea that some of the benefits of CR could be recapitulated without restricting caloric intake *per se*. Furthermore, despite the potential benefits of CR for improving CV function with aging, most older adults are not willing to practice sustained CR. Sustained CR also poses a potential risk to normal-weight older adults who are already prone to age-associated loss of bone density^[188]^ and skeletal muscle mass^[189]^. Thus, the CV benefits of CR may be outweighed by these potentially adverse effects in normal-weight older adults and as such, more practical strategies are needed. Much attention has been devoted to “CR-mimicking” interventions which leverage the benefits of CR while limiting the risk of adverse side effects (previously reviewed in detail elsewhere^[190]^). One such approach has been “intermittent fasting” where energy intake (with or without CR) is limited to certain times of the day or particular days of the week (i.e., consume calories for 5 consecutive days and fast for 2 days).

We recently completed a randomized controlled trial of intermittent fasting, in which ML/O adults underwent time restricted feeding (TRF; daily 8 h feeding window starting between 10:00–11:00 AM for 6 weeks) without CR. We found that TRF was both safe (no loss of bone or skeletal muscle mass) and feasible (high adherence) in ML/O adults and improved markers of physical capacity (e.g., 6-min walk distance; lower heart rate during light and moderate intensity exercise). However, we observed no effects on CV function, which may have been due to the short duration of the intervention period^[191]^ or that TRF was done without restricting overall caloric intake^[192,193]^. Nonetheless, these findings may serve as a foundation for future TRF trials with longer duration and/or the use of other CR-like interventions (i.e., interventions that target deregulated nutrient sensing).

Other dietary approaches that have been associated, at least in part, with healthy aging include branched-chain amino acid, methionine^[194]^ and protein^[195,196]^ restriction, and a ketogenic diet^[197]^. The putative mechanisms underlying the healthy aging-associated effects of these dietary strategies are a reduction in oxidative stress and inflammation, which appear to be related to reduced activation of mTOR and the CV aging hallmarks “deregulated nutrient sensing” and “impaired protein homeostasis”^[194–196]^. However, to date, there have not been any RCTs directly assessing the influence of these dietary approaches on CV function in ML/O adults, which is highlighted further in the “Research Gaps and Future Directions” section.

#### Dietary sodium restriction

Considering the clear link between excess dietary sodium intake and CV dysfunction^[198]^, reduced sodium intake is recommended as a lifestyle/nutrition strategy for individuals at increased risk for developing CVD (e.g., ML/O adults). Historically, dietary sodium restriction (DSR) had been advanced as a therapeutic strategy for reducing BP [e.g., a significant component of the dietary approach to stop hypertension (DASH) diet^[199]^]; however, excess dietary sodium intake has adverse effects independent of BP. Preclinical animal studies^[200,201]^ and human cross-sectional investigations^[202]^ have demonstrated an inverse relation between dietary sodium intake and vascular endothelial function. To extend these observations, our group performed a 4-week crossover trial in which ML/O adults consumed either 1.2 g of sodium per day (DSR; amount of sodium recommended in the DASH diet^[199]^) or control 3.6 g of sodium per day (national average of sodium intake per the National Health and Nutrition Examination Survey^[203]^), and then crossed over to the opposite condition. We found that DSR could improve both conduit and resistance artery endothelial function by increasing NO bioavailability secondary to reducing excess oxidative stress-related suppression of endothelial function^[204]^. Furthermore, we also have shown that DSR is associated with reduced aortic stiffness in ML/O adults^[205,206]^. Building from our findings, recent evidence from others suggests that DSR may improve endothelial function by altering arteriolar DNA methylation patterns (i.e., epigenetics)^[207]^. Taken together, DSR may be a safe and effective dietary strategy for improving CV health in ML/O adults, but more work is needed to determine the mechanisms of action of DSR, including if it influences hallmarks of CV aging, in addition to influencing oxidative stress (highlighted in the research gaps and future directions section) [Figure 5].

#### Other healthy dietary patterns

As previously mentioned, the DASH diet was specifically designed to target BP, and its efficacy for reducing BP in ML/O adults is well established. Interestingly, recent evidence from a large cohort longitudinal study also suggests that the DASH diet is independently associated with an attenuated increase in arterial stiffness between the ages of 36 and 64 years^[208]^; however, the influence of the DASH diet on endothelial function is mixed^[209,210]^. Furthermore, it is currently unclear whether the DASH diet could reverse established vascular dysfunction in ML/O adults (discussed further in the research gaps and future direction section).

The Mediterranean diet is defined as a diet rich in whole grains, vegetables, legumes, fruits, seeds, herbs, spices, olive oil, and in moderation, seafood, dairy, and poultry. The Mediterranean diet has shown clear efficacy for improving arterial stiffness, endothelial function, SBP^[211,212]^, and preventing CVD^[213]^; however, only the effects on arterial stiffness and SBP have been established in older adults^[212]^, suggesting future studies are warranted to determine the influence of the Mediterranean diet on endothelial function in ML/O adults [Figure 5].

The CV function-promoting effects of both the DASH and Mediterranean diets are thought to be due, in part, to the greater soluble fiber intake that accompanies these dietary patterns. Multiple epidemiological and large-cohort intervention studies have shown that high-fiber diets reduce CVD-related morbidity and mortality^[214–217]^, as well as the prevalence of CVD risk factors^[218]^. Unfortunately, a very low percentage of ML/O adults meet the recommended daily intake for total dietary fiber (25–30 g/day)^[218]^, and as such, the DASH and Mediterranean-style dietary patterns may represent effective approaches for increasing total fiber intake in ML/O adults, although adherence is still a major concern. To isolate the effect of dietary fiber on arterial function in ML/O, our group recently conducted a controlled feeding trial in ML/O adults (NCT03334201), in which one arm of the study included a short-term (7-day) high-fiber diet. Findings from this study suggest that increasing dietary fiber in ML/O adults is a safe and effective strategy for improving endothelial function and SBP (reduced by 4 mmHg in 7 days), and that these improvements may be due to a reduction in vascular oxidative stress^[219]^ [Figure 5]. However, more work is needed to determine if high fiber diets directly impact vascular function with aging and the potential mechanisms underlying these effects.

### Nutraceuticals and synthetic pharmacological agents

Regular aerobic exercise, healthy dietary patterns, energy restriction (when appropriate), and specific nutrient restriction (e.g., DSR) are likely to be the most robust approaches for promoting healthy CV aging. From a public health perspective, these interventions should be considered as “first-line” strategies^[220,221]^. However, there are several reasons why ML/O adults may not adopt these dietary patterns or lifestyle behaviors - e.g., poor education, social/lifestyle factors, inability to adhere, and cost. As a result, there is significant interest in evidence-based treatments that may mimic CV health-promoting effects of healthy lifestyle practices. One possibility could be “drug repurposing”, such that a drug used to treat hypertension in ML/O adults may also reduce arterial stiffness^[222]^ or a drug used to target inflammation could treat age-related endothelial dysfunction^[45]^. However, an alternative approach could be the use of “natural” treatments, such as nutraceuticals, defined as individual food ingredients/constituents with bioactive properties that may benefit human health (or in this case, CV aging^[7,223]^) or certain lifestyle-inspired synthetic pharmacological agents with favorable safety profiles.

**Figure 6.**
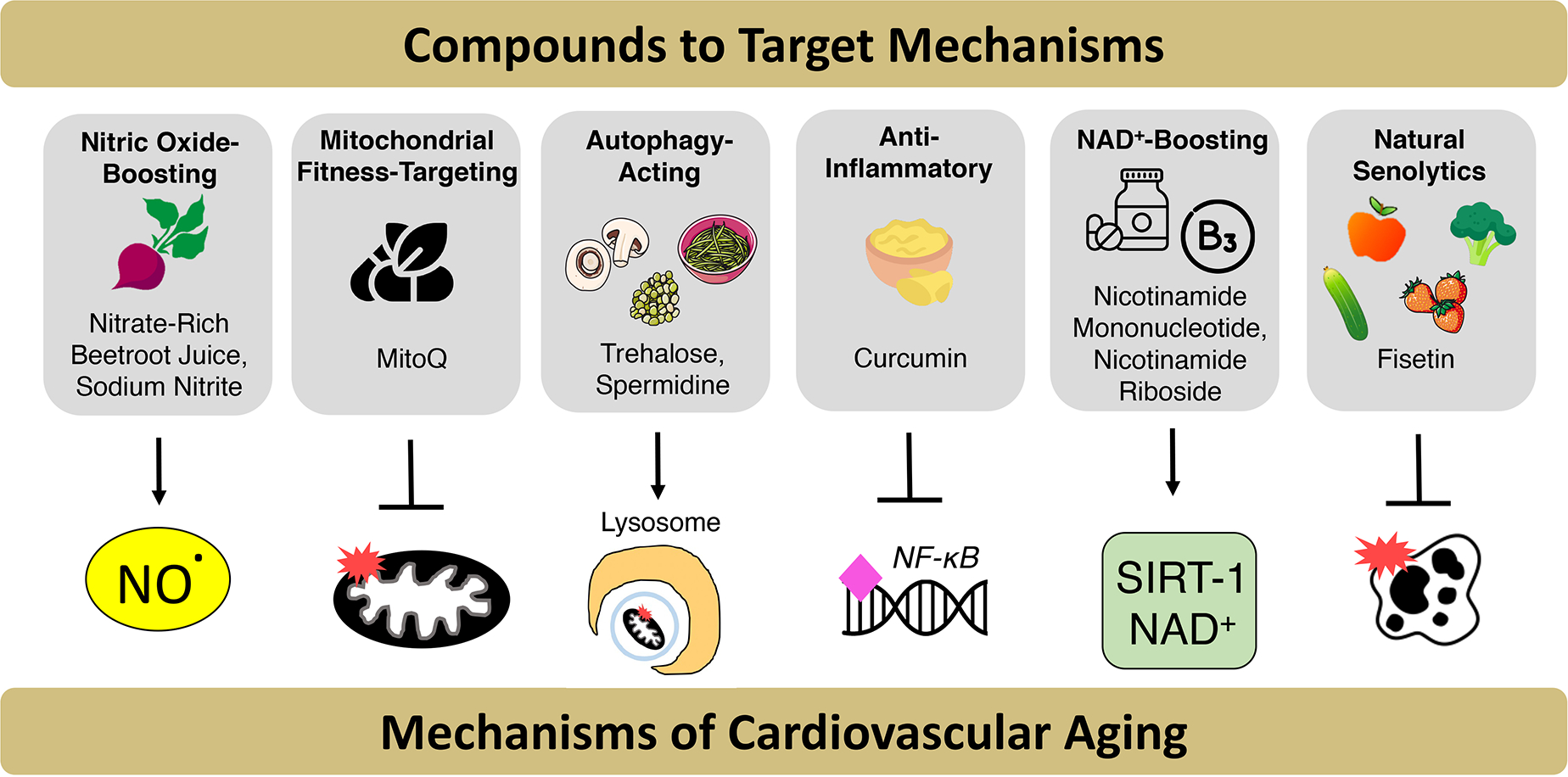
Nutraceuticals and synthetic pharmacological agents that target mechanisms of cardiovascular aging. Due to barriers that may hinder adherence to consuming a healthy diet and aerobic exercise, nutraceutical and pharmacological compounds that mimic the benefits of “first-line” cardiovascular (CV) health-promoting behaviors are being studied as alternatives to promote optimal CV aging. These compounds target mechanisms of CV aging to improve overall vascular function, including (1) Reduced nitric oxide (NO) bioavailability; (2) decreased mitochondrial fitness; and (3) autophagy; (4) increased inflammation; (5) reduced NAD^+^ bioavailability; and (6) increased cellular senescence.

Over the last ~11 years, we have taken a significant interest in nutraceuticals and other compounds that may target the primary mechanisms of age-associated vascular dysfunction, including the hallmarks of CV aging. In the remainder of this sub-section, we will highlight our work on compounds that target: (i) NO bioavailability; (ii) mitochondrial health/fitness, mitophagy and autophagy; (iii) inflammation; (iv) energy sensing and/or NAD^+^ homeostasis; and (v) cellular senescence [Figure 6].

#### NO bioavailability

As previously stated, NO bioavailability is reduced with advancing age and can be increased with certain lifestyle interventions^[9]^. Thus, NO represents a viable therapeutic target for treating and/or preventing CV aging^[224]^. Interventions seeking to enhance NO production via eNOS, including increasing L-arginine - the substrate for NO production by eNOS - have been largely ineffective in improving vascular function in healthy ML/O adults^[224]^. Thus, we sought to target NO through the eNOS-independent nitrate-nitrite-NO pathway^[225]^. We first performed an oral supplementation (drinking water) study with inorganic nitrite, in which inorganic nitrite, in the form of sodium nitrite (50 mg/L), was administered for 3 weeks to old and young mice. We found that inorganic nitrite supplementation completely reversed age-related endothelial dysfunction by increasing NO bioavailability, secondary to an amelioration of oxidative stress, and reduced arterial stiffness^[226]^. Using a similar study design, we determined that inorganic nitrite-mediated aortic de-stiffening occurred as a result of normalization (back to young levels) of aortic AGEs^[227]^. We then translated our findings in old mice to humans by conducting a small pilot clinical trial, in which ML/O adults consumed 80 or 160 mg/day of sodium nitrite (or placebo) for 10 weeks. We found that those consuming sodium nitrite had improved endothelial function and reduced arterial stiffness (observed with stiffness index - a largely BP-independent measure of arterial stiffness - but not PWV or carotid compliance)^[119]^, without clear dose-dependent effects. Next, we further translated these findings to a large multi-year RCT in which ML/O adults received 80 mg/day of sodium nitrite for 12 weeks, and confirmed the efficacy of sodium nitrite for improving endothelial function^[121]^, but did not assess arterial stiffness given the lack of clear effects in the pilot study.

In our large RCT with sodium nitrite, we obtained novel insight regarding the mechanisms by which sodium nitrite improves endothelial function by assessing the influence of nitrite treatment on mitochondrial fitness. We first showed that changes in circulating factors in plasma from subjects treated with sodium nitrite decrease mitochondrial ROS in endothelial cells in culture. We then employed a reverse translational study design to determine the functional role of suppression of mitochondrial ROS in mediating sodium nitrite-induced improvements in endothelial function with aging. To accomplish this, we administered sodium nitrite to young and old mice (as described above) and determined that sodium nitrite improved endothelial function with aging by ameliorating excess mitochondrial ROS and by increasing vascular mitochondrial stress resistance^[121]^. Collectively, these results suggest that enhancing NO bioavailability by targeting the nitrate-nitrite-NO pathway may be a viable therapeutic approach for improving CV health with aging, in part by altering circulating factors and improving mitochondrial health/function. We are now further translating these findings by performing a multi-year RCT with inorganic nitrate supplementation via nitrate-rich beetroot juice to enhance NO bioavailability for improving vascular function and BP in patients with CKD (NCT03826147). If shown to be safe and effective, inorganic nitrate-rich beetroot juice could be a highly useful nutraceutical for improving vascular function and reducing the risk of CVD in CKD, as CVD are a leading cause of death in this patient population^[228]^ [Figure 6].

#### Mitochondrial health/fitness, mitophagy and autophagy

As previously mentioned, mitochondria are a key source of excess oxidative stress with aging (as a result of excess ROS bioactivity and a reduction in mitochondrial antioxidant defenses - e.g., MnSOD). Thus, targeting mitochondrial health/fitness is emerging as an effective therapeutic approach for preventing and treating age-related vascular dysfunction, which we have reviewed in detail elsewhere^[38]^.

For example, we have shown that supplementing old mice with the mitochondria-targeted antioxidant MitoQ (250 μM in the drinking water for 4 weeks) completely restores (back to young levels) endothelial function by increasing NO bioavailability, decreasing mitochondrial ROS and, in part, by increasing mitochondrial stress resistance - i.e., MitoQ treatment ameliorates the acute reduction in endothelial function observed when vessels from old mice are exposed to a mitochondrial stressor. Furthermore, these effects were associated with the restored abundance of MnSOD and favorable changes in markers of mitochondrial abundance and homeostasis^[40]^. We then translated these preclinical findings to humans in a pilot, placebo-controlled, crossover design clinical trial in ML/O adults and demonstrated that MitoQ supplementation (20 mg/day for 6 weeks) increased NO-mediated endothelial function, which occurred, in part, by ameliorating excess mitochondrial ROS^[229]^. Our initial observations also suggested that MitoQ lowered aortic stiffness (in subjects with marked elevation in arterial stiffness at baseline). We are now aiming to extend these findings to a larger properly powered cohort of ML/O adults in a 12-week parallelarm placebo-controlled RCT to establish the efficacy of MitoQ supplementation for improving vascular function and determine the associated mechanisms of action (NCT04851288) [Figure 6].

To target reductions in mitochondrial quality control with aging, we supplemented old mice with the disaccharide and natural autophagy enhancer, trehalose (2% in the drinking water for 4 weeks) and found that trehalose could reverse age-related endothelial dysfunction by enhancing NO bioavailability and ameliorating excess ROS-related suppression of EDD and could reduce age-related arterial stiffness. These changes were associated with reduced inflammation and increased activation of mitochondrial quality control pathways and autophagy in the vasculature^[90,92]^. Furthermore, trehalose reduced vascular mitochondrial oxidative stress, indicating increased mitochondrial health^[92]^. We next translated these findings to ML/O adults and found that oral supplementation with trehalose (for 12 weeks) could improve resistance artery EDD by increasing NO bioavailability and ameliorating excess oxidative stress; however, trehalose did not influence arterial stiffness in this cohort^[230]^. Given that trehalose is a disaccharide (carbohydrate), chronic supplementation resulted in a gain in body weight^[230]^, which ultimately limited its future translatability, given the adverse influence of excess body weight on vascular function^[185]^.

We have also targeted autophagy with the natural polyamine spermidine and found that spermidine was generally safe and could restore age-related impairments in NO-mediated endothelial function and reverse aortic stiffening in mice^[231]^. The improvements in vascular function were mediated by reductions in vascular oxidative stress and associated with increased activation of autophagic pathways^[231]^. These preclinical findings have yet to be translated to humans, but dietary spermidine intake has been inversely associated with BP and risk of CVD^[232]^ and spermidine supplementation has been shown by others to be safe and welltolerated in older adults^[233]^ [Figure 6]. Thus, it is warranted to perform a properly powered clinical trial aimed at determining the influence of spermidine supplementation on improving vascular function with aging.

#### Inflammation

As previously discussed, inflammation is a well-established mechanism of CV aging^[24,42]^. As such, we have sought to determine the role of the well-established anti-inflammatory nutraceutical curcumin (the primary polyphenol found in turmeric) in treating vascular aging. We found that 4 weeks of oral curcumin supplementation (0.2% in the drinking water) could completely restore NO-mediated endothelial function and normalize arterial stiffness in old mice by ameliorating excess oxidative stress^[234]^. We next conducted a pilot clinical trial in ML/O adults and found that 12 weeks of oral curcumin supplementation (2 g/day) could improve NO-mediated resistance and conduit artery endothelial function by reducing excess oxidative stress, but there was no influence on arterial stiffness^[235]^. We (as co-investigators) are now translating these findings to patients with CKD (NCT03223883); however, curcumin did not influence vascular function in patients with autosomal dominant polycystic kidney disease^[236]^ [Figure 6]. Taken together, curcumin may be a viable nutraceutical for the treatment of vascular dysfunction in other patient populations of accelerated vascular aging (highlighted further in the research gaps and future directions section).

#### NAD^+^ bioavailability

Reduced NAD^+^ bioavailability is thought to be an important mechanism of age-related declines in physiological function, in part due to the role of NAD^+^ as a substrate for SIRT-1 and its association with deregulated nutrient sensing. NAD^+^ is also an essential coenzyme for a variety of cellular reactions that regulate DNA repair, inflammation, metabolism and other processes^[103]^. Therefore, supplementation with precursors for NAD^+^ biosynthesis has become one of the most popular ways to attempt to boost NAD^+^ and promote healthy aging. Enhancing NAD^+^ bioavailability is a compelling therapeutic approach for treating and/or preventing vascular dysfunction with aging given that increasing SIRT-1 abundance/activity is an effective strategy for improving vascular function with aging^[101]^. Indeed, we found that oral administration of the NAD^+^ precursor, nicotinamide mononucleotide (NMN; 300 mg/kg/day for 8 weeks in the drinking water) could restore NO-mediated endothelial function by ameliorating excess whole-cell ROS bioactivity and reverse arterial stiffness by favorably modulating aortic intrinsic mechanical wall stiffness and abundance of structural proteins (i.e., greater elastin and lower collagen) in old mice^[237]^. Old mice that received NMN supplementation had a greater abundance of SIRT-1 and reduced acetylation of NF-κB (downstream target of SIRT-1, indicating increased SIRT-1 activation) in the vasculature, suggesting that enhancing NAD^+^ with NMN may reduce inflammation by modulating SIRT-1 to improve vascular function with aging^[237]^.

We next conducted a pilot randomized, double-blinded, placebo-controlled crossover design clinical trial in ML/O adults using another NAD^+^ precursor, nicotinamide riboside (NR). In this study, we demonstrated that NR (1000 mg/day for 6 weeks) was safe and increased NAD^+^ bioavailability, indicated by an increase in NAD^+^ metabolites in peripheral blood mononuclear cells. We also found that NR could lower SBP by 4 mmHg and decrease arterial stiffness (PWV), with particular efficacy (9 mmHg reduction in SBP) in those with above-normal SBP (i.e., SBP ≥120 mmHg) at baseline^[238]^. To further translate these findings, we are now conducting a multi-year large-scale placebo-controlled RCT with NR in ML/O adults with above-normal SBP (NCT03821623^[239]^) [Figure 6].

#### Cellular senescence

Cellular senescence has become one of the foremost targeted basic aging mechanisms for improving various age-related conditions^[240]^. Given that cellular senescence is emerging as a fundamental mechanism underlying vascular aging, targeting cell senescence to treat CV aging holds significant promise^[97–99,241]^. The current most well-accepted approach for decreasing senescent cell burden is treatment with compounds that selectively clear/kill senescent cells, termed senolytics. Senolytic compounds are typically administered intermittently, with the goal of acutely clearing excess senescent cells, while maintaining the basal cellular senescence response and not interfering with processes such as wound healing^[93]^ and suppression of cancer^[94]^.

Currently, synthetic pharmacological senolytics - (e.g., dasatinib + quercetin) are advancing to clinical trials to treat conditions such as diabetic kidney disease^[242]^ and idiopathic pulmonary fibrosis^[243]^. Unfortunately, dasatinib is a chemotherapeutic agent with significant side effects, which considerably hinders the large-scale translatability of this senolytic strategy to ML/O adults free from clinical diseases due to safety concerns. Accordingly, we are highly interested in determining the safety and efficacy of *natural* senolytics for improving age-associated vascular dysfunction, as we believe that these compounds have the greatest potential for translation to healthy CV aging. An emerging candidate for natural senolytic is the flavonoid fisetin, which has been shown to exert senolytic effects *in vivo* and to increase lifespan and markers of healthspan in mouse models of both chronological and accelerated aging^[244]^. Our early observations in old mice suggest that intermittent oral fisetin supplementation (100 mg/kg/day by oral gavage; 1 week on – 2 weeks off – 1 week on) is effective at increasing NO-mediated endothelial function and lowering arterial stiffness, and that these improvements occur as a result of suppressing excess cellular senescence in the vasculature^[245]^ [Figure 6]. However, it remains to be determined if intermittent fisetin supplementation can reduce cellular senescence and improve vascular function in ML/O adults.

## CONCLUSION, RESEARCH GAPS AND FUTURE DIRECTIONS

**Figure 7.**
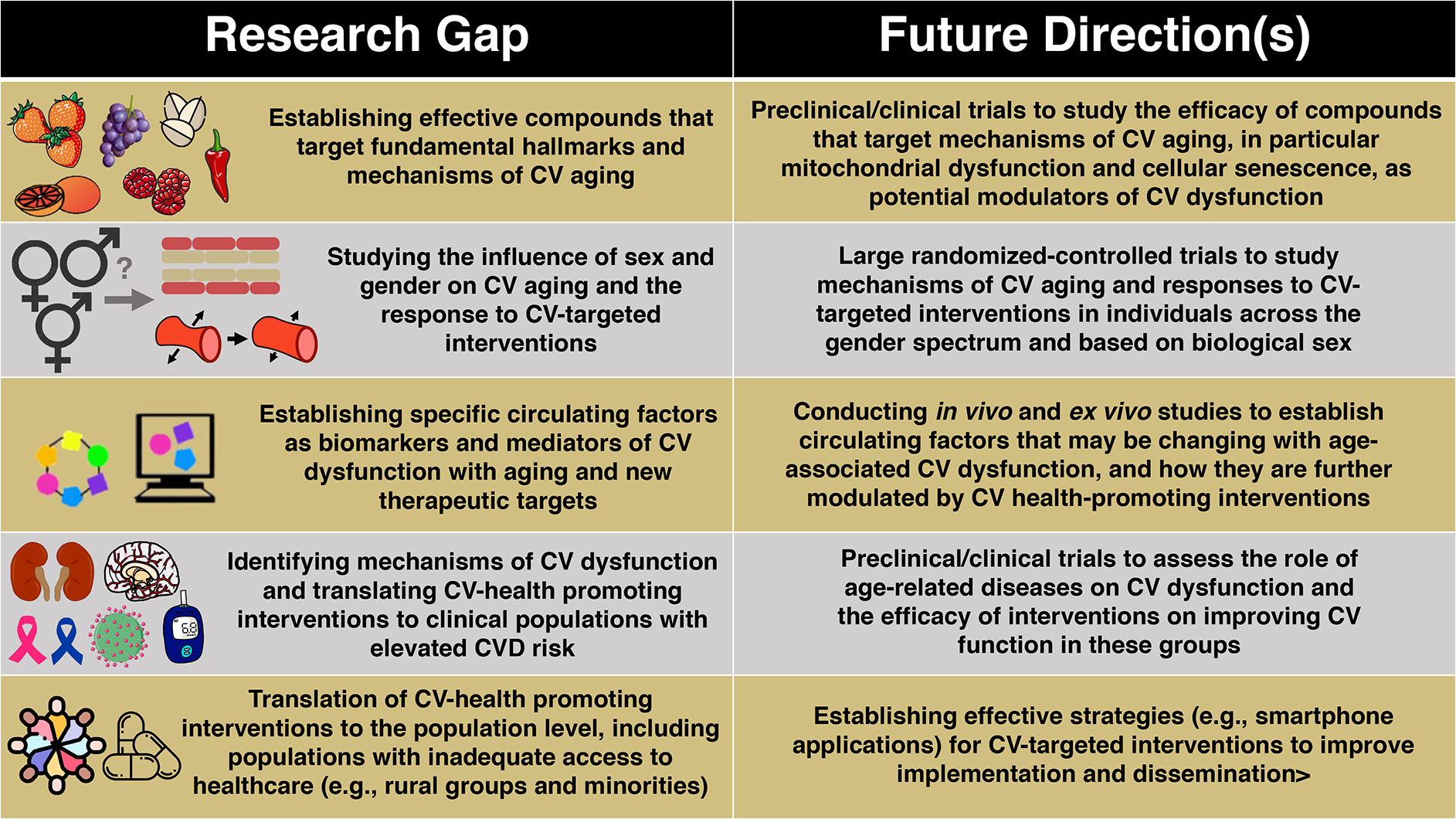
Research gaps and future directions for promoting healthy cardiovascular aging. Although there has been extensive research on the mechanisms leading to cardiovascular (CV) dysfunction and the strategies that promote healthy CV aging, several important gaps remain that should be further explored. These include, but are not limited to: (1) Establishing new and effective compounds that target mechanisms of CV aging; (2) studying the role of sex and gender on CV aging; (3) identifying novel circulating factors as biomarkers or therapeutic targets; (4) identifying mechanisms and translating effective strategies to ameliorate CV dysfunction in clinical populations that exhibit increased risk for CVD; and (5) translating effective implementation strategies for CV-targeted interventions to improve public health.

Aging is the major risk factor for CVD due importantly to the development of vascular dysfunction (in particular endothelial dysfunction and large elastic artery stiffening) and increases in SBP. In this review, we discussed fundamental mechanisms of CV aging - processes that could serve as viable therapeutic targets for the prevention and/or treatment of CV dysfunction with aging to reduce the risk of CVD. We have also reviewed how particular exercise and “exercise-mimicking” strategies can improve CV function with aging, at least in part, by targeting the hallmarks of CV aging. Finally, we highlight established and emerging dietary patterns, nutraceuticals and synthetic pharmacological compounds that may serve as healthy CV aging interventions. There remain several important knowledge gaps in the field; the following represent some potential future biomedically significant directions for research related to healthy CV aging [Figure 7].

### Establishing novel nutraceuticals for targeting fundamental mechanisms of CV aging

Determining the CV health promoting effects of nutraceuticals for targeting the biological hallmarks of aging. For example, nutraceuticals that target mitochondria (e.g., mitophagy activation with urolithin A) and natural food extracts that target cellular senescence (e.g., extracts or analogs of grapeseeds^[246]^, oats^[247]^, peppers^[248]^, ginger^[249]^, and curcumin^[250]^) or other select natural compounds (e.g., 25-hydroxycholesterol^[251]^).

### Assessing and addressing biological sex, transgender and gender diversity as variables that influence CV health and response to healthy CV aging interventions

A better understanding of sex and sex hormones as biological variables underlying CV aging must be determined^[139,141,146]^. In addition, we need to better understand transgender and gender diversity as variables that influence CV health, how transgender and gender diversity may influence the response to healthy CV aging interventions, and how sex hormones affect people of differing biological sexes^[252]^. Appropriate preclinical approaches and experiments and inclusion of these groups in large, properly-powered clinical trials are needed to accomplish this goal^[253]^.

### Role of circulating factors

Discovering novel circulating factors that are altered with aging and significantly associated with CV dysfunction is a necessary next step to developing validated biomarkers of CV aging^[22]^. Furthermore, establishing the direct cause-and-effect role of these circulating factors on CV function would establish these circulating factors as new therapeutic targets to promote healthy CV aging. In addition, determining which circulating factors are changed with healthy CV aging-promoting interventions^[120–122]^ would lend additional mechanistic insight into how these interventions are transducing beneficial effects.

### Translation of promising interventions to other populations characterized by accelerated CV aging and elevated disease risk

Promising strategies for improving CV health with aging should be translated to other populations characterized by accelerated vascular aging as a result of clinical disease (e.g., CKD^[57,63]^, Type 2 Diabetes^[64,65]^, COVID-19^[254–257]^) and/or disease treatment (e.g., chemotherapy-treated cancer survivors^[258]^, patients living with HIV on antiretroviral therapy^[259]^). Furthermore, there is an increasing body of literature suggesting that Alzheimer’s disease and related dementias manifest partly as a result of CV dysfunction^[57,260]^; as such, cognitive and/or cerebrovascular function should be assessed in parallel with peripheral CV function. Moreover, a better understanding of how fundamental mechanisms of CV aging underly CV dysfunction in populations with accelerated aging could inform future therapeutic strategies in these groups.

### Public health translation of healthy CV aging interventions

Efforts must be made to translate CV aging interventions that have been shown to have therapeutic efficacy (i.e., improve CV function under highly controlled conditions) for use in the public health domain. Steps along this translational research spectrum that remain to be made include establishing large-scale therapeutic efficacy (improving CV health in real-world settings) and developing methods for effective dissemination and implementation (promoting intervention adoption and adherence). Of particular importance will be reaching underserved groups, such as low socioeconomic status individuals or rural communities without access to healthcare infrastructure. Technology-based approaches, such as smartphone application-delivered healthy lifestyle approaches, are particularly promising for achieving these public health translation goals. Of the interventions discussed above, high-resistance IMST is an example of an efficacious healthy CV aging intervention that may be ready for public health translation efforts^[171]^. Overall, effective dissemination and implementation of promising, evidence-based interventions, are crucial for promoting healthy CV aging.
